# Electroencephalographic features of the developing brain in 72 dogs under xylazine sedation: a visual and statistical analysis

**DOI:** 10.3389/fvets.2023.1150617

**Published:** 2023-06-29

**Authors:** Fernando Pellegrino, Christian M. Gómez Álvarez

**Affiliations:** ^1^Anatomy Department, Facultad de Veterinaria, Universidad de Buenos Aires, Buenos Aires, Argentina; ^2^Neurophysiology and Clinical Neurology Service at VetCam Specialists, Valencia, Spain

**Keywords:** electroencephalogram, developing brain, brain maturation and development, normal electroencephalogram, rhythm, amplitude-frequency characteristics

## Abstract

Electroencephalogram (EEG) is a neurophysiological test, which is widely used in human medicine for epilepsy diagnosis and other neurological disorders. For an adequate interpretation, it is necessary to know the electroencephalogram features for different stages of development. Despite the growing interest in its implementation in veterinary medicine, standardized descriptions of the EEG features of the different stages of brain development in dogs are restricted to studies with limited number of dogs and limited age groups. In this research, the electroencephalographic recording of 72 dogs of different breeds and ages was carried out under xylazine sedation to determine tracing characteristics by visual analysis and through statistical analysis of power spectrum. To establish the EEG features of recordings, 3 essential aspects were selected: (a) the presence or absence of slow waves of 4 to 6–7 Hz; (b) the comparison of the electrical activity recorded in the temporal and dorsal cortex channels; and (c) the visual increase of the alpha activity. Visual analysis on both reference and bipolar montage was performed by the authors and additionally blindly corroborated by two human neurophysiologists. The results allowed us to differentiate 5 age groups: 0–5, 6–11, 12–17, 18–23, and >24 months. Statistical analysis of the power spectrum was performed by analysis of variance (ANOVA) with a completely randomized design (CRD) under factorial arrangement by observing the effect of ages, channels and electroencephalographic rhythms on relative power. The results obtained matched those observed in the visual analysis. According to our results, the characteristics of the EEG corresponding to the adult animal begin to appear at 12 months of age but stabilize after 24 months of age. In this case, the evident differences in the processes of development and maturation of the neopallium and the rhinencephalon play a determining role. Our results differ from those obtained by other authors, probably due to the addition of a deep electrode that facilitates the recording of temporal cortical activity and its deeper rhinencephalic connections.

## 1. Introduction

Encephalographic recordings in normal adult dogs depending on the state of arousal includes, mainly, 6 to 12-Hz waves (theta and alpha activity type), with a predominant alpha rhythm. This activity may be recognized all over the brain cortex, but mostly, in parietal and occipital regions of mesocephalic and dolichocephalic dogs, and parietal and frontal regions of brachycephalic dogs. The recording amplitude varies among animals, probably depending on the distance between electrodes and the layers of active dipoles, which are proportional to skull's thickness and, therefore, to the distance between the active zone and the recording electrode, but always lower at the frontopolar and temporal areas in all breeds (1).

There is considerable disparity of opinion regarding age-related variations in electroencephalograms (EEG) in dogs, particularly concerning the maturation period. Charles and Fuller reported that the EEG of an 8-week-old puppy is similar to that of an adult dog (2). Petersen et al. (3) described that at 4 weeks of age, the EEG shows a maturity pattern that evolves until remaining constant after 7 weeks of age. Although Fox does not establish in detail the maturation period of the electroencephalographic recording, he states that there exists a significant amplitude decrease between 12 and 16 weeks of age, while the frequency progressively increases until stabilizing at adult age (4–6). Results of inter- and intra-hemispheric coherence studies, according to which all the neocortex takes a similar phase interaction between 6 and 14 weeks of age, seem to support the prior statements (7, 8). Redding claims that EEG maturation develops between 22 and 30 weeks of age, depending on gender and breed (9). Pampiglione's research about brain electrical activity in young dogs indicates that from the 6th month, the recording acquires the features that persist in the adult animal (10). Senba et al. define the immaturity period as the one during which new waveforms and rhythms are observed. According to that criterion, they suggest that the maturation period may take place between 5 and 6 weeks of age, a time when the stabilization of dendritic arborization and an increase of the cortex metabolic rate ensue. However, in the same study, and based on waves amplitude and frequency, they believe maturation may also take place between 20 and 30 weeks of age. Such statements arise from evaluations performed on Beagle dogs, in litters that were followed up from birth to 50 weeks of age (11).

Discrepancies are probably the result of the different criteria when defining the changes found on EEG, during the transition from puppy to adult age. However, from a clinical point of view, EEG maturity has been established in dogs at the age of 6 months, with a predominance of slow waves in the previous stage (12, 13). After the age of 1 year, no research has shown differences in the electroencephalographic tracing between puppies and young or adult dogs, although it has been stated that a decrease in amplitude can be observed in old dogs.

This study's objective was to describe the normal background activity and superimposed transients in dogs of different ages using 10 dorsal subcutaneous electrodes and two deep lateral electrodes that allowed recording the activity of temporal cortex under xylazine sedation. A visual analysis of recordings was achieved by four different researchers and statistical analysis of the relative potency was also done.

## 2. Materials and methods

This is an observational and prospective study. Therefore, live animals were used in this study, and ethical approval was granted from Comisión Institucional para el Cuidado y Uso de Animales de Laboratorio (CICUAL) and Facultad de Ciencias Veterinarias-Universidad de Buenos Aires. All owners of the animals included in this study, received informed consent, and had approved that their dogs and data would be used for academic research.

### 2.1. Population

Seventy two healthy dogs with unremarkable neurological and physical exam were included. Neither previous infectious nor neurological diseases were reported. Dogs were included regardless of gender or breed and was based on previous studies indicating that the frequency and electrical characteristics of brain waves are comparable in any breed, although their amplitude is not (9, 14). Dogs with any neurological symptom, any physical exam or blood workout abnormalities were excluded. Hypothetical differences of EEG maturation among dog breeds were not considered, as their structural development is similar; should there exist a difference, it would not depend on the breed, but rather on the training and learning processes that shape intraneuronal synaptic connections (15, 16). To obtain final sample of dogs (72 dogs) we recruited 120 patient who visit our clinic, met the inclusion criteria, and could be distributed among the age groups. Then, 72 dogs (12 for each age group) were selected aleatory through a random number generator from the 120 original dogs recruited. Those patients received in exchange for allow us to do the EEG some compensation as medical attention or vaccines, and tutors signed up an informed consent. The number of dogs was appropriate to ensure a significance level of 5% and a statistical power of at least 80%.

### 2.2. Electrodes

Both for exploration and reference, 15-mm length stainless steel needle electrodes were used, (Akonic S.A., subdermal needle electrodes) (17). Electromyographic (EMG) monopolar needles (Akonic, S.A.) coated with Teflon, except for their tips, were used to record temporal region activity (T3 and T4). The length of the bare tip was 4 mm; the full length of the needle was 350 mm. This type of electrodes made it possible to go through the muscle and reach the bone, thus avoiding interferences caused by muscle activity. Impedance was measured prior to obtaining the EEGs, always resulting in about 5 kΩ. Cross-impedance or artifacts in recordings that combined subdermal needle electrodes with EMG electrodes (bipolar montage) were not found (1, 18).

### 2.3. Visual analysis

Visual analysis was performed by the authors and by two human neurophysiologists who received the recording without signalment of dogs to ensure their participation as blind reviewers. The background activity was described (rhythms of similar shape and duration, with regular and recurrent appearance) according to reviewers consensus. In addition, we describe the transient activity present in the recordings (4–6 Hz slow waves, vertex sharp waves, 3 Hz slow waves and K complexes). The criteria to determine the electroencephalographic features of transient activity in the different age groups were: (1) Grapholements were present in at least 75% (*n* = 9) of dogs in each group. (2) Graphoelements repeated with a frequency of at least once per minute, (3) All reviewers agreed on the visual findings.

### 2.4. Equipment

In order to collect the electroencephalographic recordings, a program specifically designed for computerized electroencephalography and brain mapping reconstruction, with 12 simultaneous recording channels (AKONIC BIO-PC adjusted version 7.0) was used. Technical recording parameters were as follows: common mode rejection >100 db; frequency response of 0.5 to 128 Hz; antialiasing filter: 64 Hz; notch filter for 50 Hz activity was set; high frequency filter (HFF): 70 Hz; low frequency filter (LFF): 0.5 Hz; internal noise < 1 uV; sampling frequency: 256 s; paper speed: 30 mm/s; electrodes impedance: <5 kΩ.

### 2.5. Animal restraint

Animal restraint was achieved by subcutaneously injecting 0.5 mg/kg of xylazine (Rompun, Bayer Argentina S.A.), based on previous studies (19) and our own experience (1, 18).

### 2.6. Procedure

#### 2.6.1. Recording

**Figure 1:**
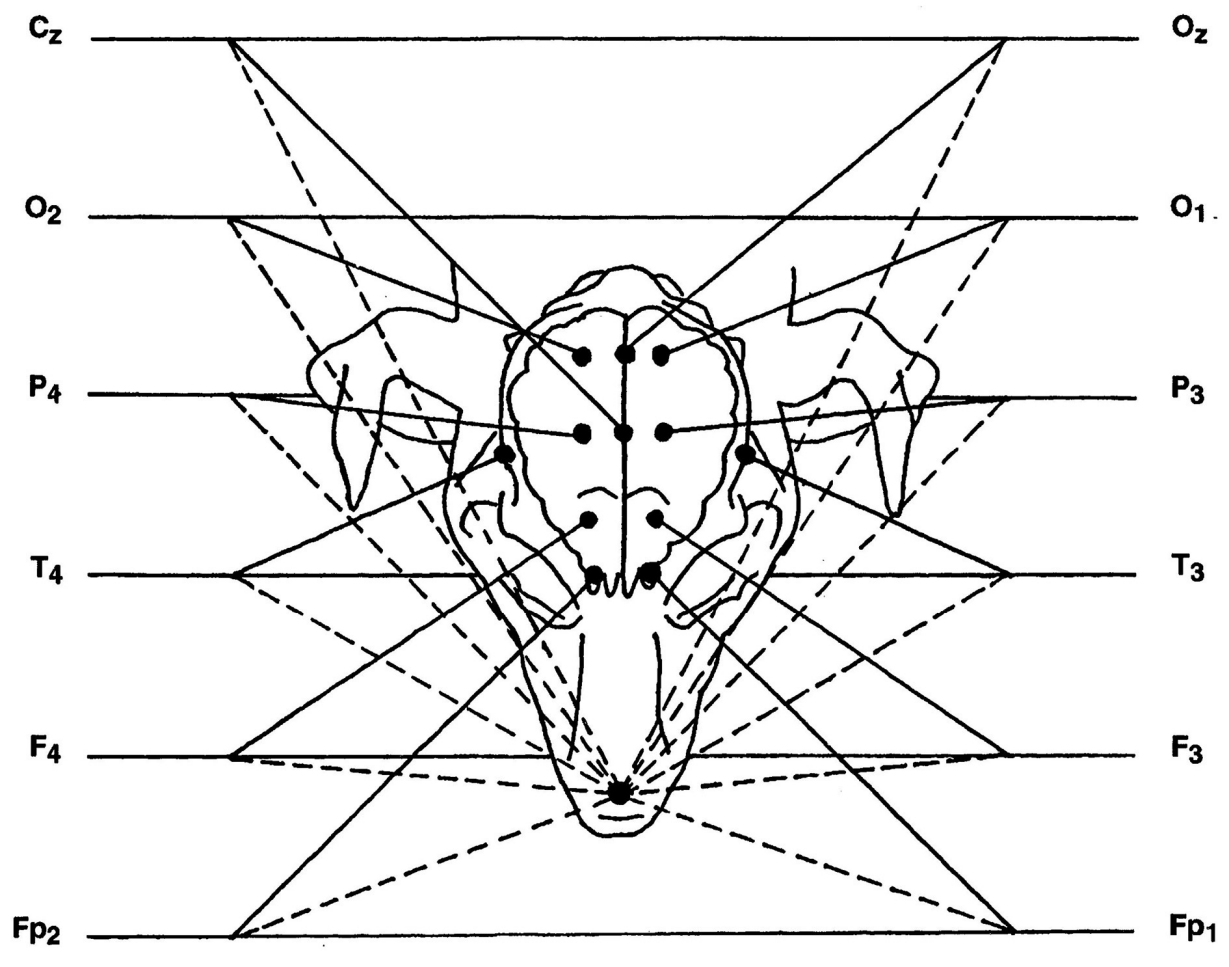
Diagram of reference montage, with nasal reference electrode. In the diagram, each channel has two inputs, represented by a solid line and a dashed line. The solid line is the input coming from the active electrode, while the dashed line is the input corresponding to the reference. Cz, Central electrode; O, occipital electrode; P, parietal electrode; T, temporal electrode; F, frontal electrode; Fp, frontopolar electrode; Oz, central occipital electrode. Even and odd numbers correspond to the right and left side, respectively.

Basic recording was done using reference montage. Active electrodes were placed at the left and right frontopolar (Fp1, and Fp2), frontal (F3, F4), parietal (P3, P4), occipital cortex (O1, O2) and temporal deep electrodes over the sphenoid complex in the ventral aspect of the lateral surface of the cranium with a reference electrode over the rhinal nasal bone. Cz, central electrode (vertex) and Oz, central occipital electrode were placed (see Figure 1). Recording was performed for 30 min (1, 18).

### 2.7. EEG quantification (mathematical analysis)

#### 2.7.1. Sample editing

For analysis purposes, stationary segments of a 2-s length (epochs) were selected, free from artifacts and transient events, averaging results of each epoch. The minimum acceptable time for averaging, from the EEG stationary standpoint, was 30 s (20), therefore, at least 15 epochs were selected for each dog.

#### 2.7.2. Spectral estimate

Fast Fourier transform (FFT) was applied to selected segments. In this way, the usual EEG image is converted into a graph (power spectrum), in which frequency components are organized on the abscissae with their pertaining power on the ordinates; the area below the curve is the absolute power, which is equal to the amplitude squared. In this graph, the dominant frequency components for each channel can be visually defined (20). Frequency components were pooled into classic clinical waves: delta (0.5 to 3.5 Hz), theta (4 to 7.5 Hz), alpha (8 to 13.5 Hz) and beta (from 14 Hz on). Whilst the power spectrum contains all frequency information within a signal period, it is difficult to characterize it in order to make comparisons. As to achieve this goal, a set of parameters or variables was defined, that is to say, numerical values that may be shown in the form of tables or distributed topographically on the spot where the signal was obtained for each channel (numerical maps). In order to characterize the power spectrum, the absolute power (total spectrum area detailed by frequency bands) was taken as a parameter, and from it, the relative power per channel was calculated (frequency components percentage for each channel). The relative power per channel was selected as a parameter since it allows independence from the variability in amplitude caused by morphometric characteristics of each dog (i.e.: skull size, bone thickness, and muscle layer) (9, 14) and by extra-cerebral factors that affect physical electrode features (17). Band relative power for each channel was calculated by dividing the absolute power of each frequency band for the spectrum total area (20). For the amount of data available to us, relative power showed abnormal behavior (Shapiro-Wilk test *p* < 0.05). Therefore, it was necessary to correct it by applying a Z transformation to normality using a software to scale all measurements with normal distribution, in units proportional to probability in a range from 0 to 1. This allows calculate probability of data observed regardless of initial measurements physical dimensions. Under the same test conditions, this transformed variable exhibited a normal behavior, so statistical analysis was performed based on it (21).

### 2.8. Statistical analysis of the power spectrum

The impact of age, channels and electroencephalographic rhythms on relative power was analyzed, contrasting their averages. It was determined whether there existed an interaction among these factors and, if so, which ones were involved.

The statistical analysis of the relative power behavior was divided into two stages. During the first stage, due to the lack of previous knowledge on the features of the variables being studied, we evaluated its behavior within a pilot sample in order to estimate the definite sample size that would ensure a sufficient power when reading the tests. In order to broadly define and evaluate the behavior of relative power, we considered that apart from the different ages of individuals, the variability introduced by channels involved and by various electroencephalographic rhythms needed to be isolated. Data was subjected to an analysis of variance (ANOVA) with a completely randomized design (CRD) under factorial arrangement. This arrangement allowed us to compare the effect introduced by the different channels, ages and rhythms, testing their interactions simultaneously. Ages were grouped in four levels (from 0 to 5 months, from 6 to 11 months, from 12 to 23 months, and more than 24 months), each rhythm represented a level (alpha, beta, delta and theta) and each channel represented a level, resulting in a total of 12. The model consisted of a three-level 4 x 4 x 12 factorial arrangement (8). Since the repetitions of observations were made upon the same individuals within each age group, there was a loss of independence between the repeated observations for channels and rhythms. Hence, factorial arrangement had to be corrected into “split-plots” (22, 23), making it possible to correct the lack of independence of rhythm and channel factors. From results obtained in the first stage, it was possible to develop the experimental design for subsequent tests.

In the second stage, due to an interaction among factors (there was no triple interaction), the ANOVA with CRD was respected with an age x rhythm x channel factorial arrangement. Given its minor contribution as a source of variability, beta rhythm was excluded from the analysis. Age was subdivided in comparable space-groups (0 to 5, 6 to 11, 12 to 17, and 18 to 23 months). This new division was adopted considering that, in view of the results of the pilot test and previous biological observations, variability introduced by age should occur during the first development stages. The different channels were rearranged in two levels: one of them, represented the temporal channels; the other one, the dorsal cortex portion channels. The factorial arrangement was then set up as follows: age (four levels), waves (three levels) and channels (two levels). Results were tested according to ANOVA in CRD with a 4 × 3 × 2 factorial arrangement.

Remaining ages were analyzed separately, with the purpose of determining what the changes in power spectrum of adult and elderly animals were. Two groups were formed (24 to 83 months of age and more than 84 months of age). Factorial arrangement for this age group was set up as follows: age (two levels), rhythms (three levels) and channels (two levels). Results were tested according to ANOVA in CRD with a 2 × 3 × 2 factorial arrangement (23). In all cases, a significance level was set at a = 0.05.

Finally, all individual values corresponding to absolute power were considered and the average and standard deviation of the sample were calculated for each of the recording channels, in order to elaborate the descriptive statistics of the power spectrum. This procedure was performed for each preselected age group. Amplitude and relative power per channel were calculated from this data (square root of absolute power), obtaining the average and standard deviation as well.

## 3. Results

### 3.1. Population

Dogs were divided into six groups of 12 individuals, according to their age: (a) group 1, up to 5 months of age; four German shepherd, three mixed breeds, two French poodles, one Boxer, one Dalmatian, one Yorkshire terrier, eight females, and four males, between 1.5 to 12 Kg of body weight (BW), (b) group 2, from 6 to 11 months of age; three mixed breed, two French poodles, one Great dane, one Cocker spaniel, one Spanish Breton, one Basset hound, one German Shepherd, one Bichon frisse, and one American Pitbull. Seven females and five males, from 4.2 to 31.3 kg of BW; (c) group 3, from 12 to 17 months of age; four mixed breeds, three Cocker spaniels, one Yorkshire terrier, one French poodle, one Beagle, one Airdale terrier and one German shepherd. Seven females and five males between 3.4 and 28.9 Kg of BW; (d) group 4, from 18 to 23 months of age; three mixed breeds, two French poodle, two Doberman pinscher, one Giant schnauzer, one Cocker spaniel, one Yorkshire terrier, one Beagle, one Dogo argentino. Five females and seven males between 3.5 and 38.6 kg of BW (e) group 5, from 24 to 83 months of age; eight mixed breeds, two Siberian Husky, one Dalmatian, one Pointer. Six females and six males between 22.3 kg and 36.8 Kg of BW and (f) group 6, more than 83 months of age, nine mixed breeds, one Shih-tzu, one Boxer, and one French poodle. Eight females and four males between 4.6 and 32.6 Kg of BW. Dogs of groups 1, 2, 3, and 4 had a clinical follow-up for 1 year, with regular physical and neurological examinations every 4 months, without relevant findings suggesting a neurological disease (24).

Dogs from group 1, 2, 3, and 4 included dogs whose central nervous system (CNS) was still in the process of maturing, and the entire brain cortex was also expected to display a similar type of electrical activity. This means that as the dog growth electrodes activity recorded seems similar between them regarding frequencies and amplitude. This change is evident particularly at the temporal leads. Dogs from group 5 and 6 included dogs whose CNS was already matured, and the electroencephalogram recording looked stable (9, 11). There is no detailed investigation as to what happens in the EEG after that age, hence the age in the rest of the groups was established arbitrarily.

### 3.2. Morphology of background activity (visual analysis)

#### 3.2.1. Group 1 (up to 5 months of age)

**Figure 2:**
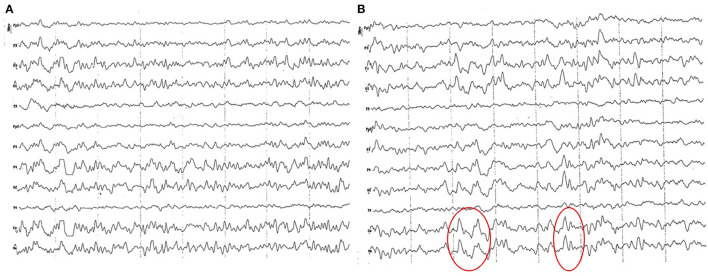
EEG (reference montage) corresponding to a female German Shepherd of 1 $\frac{1}{2}$ months of age. Each vertical division represents 1 second and the small vertical bar at the upper left corner represents 50 μV. **(A)** Predominance of slow waves can be observed, showing a very weak outline of superimposed alpha wave, of higher amplitude in the occipito-parietal channels. Temporal (T) and frontopolar (Fp) channels show a very low voltage signal, with a predominant slow activity of 3 to 4 Hz. **(B)** Sharp high voltage waves of the vertex are clearly observed (red circles).

**Figure 3:**
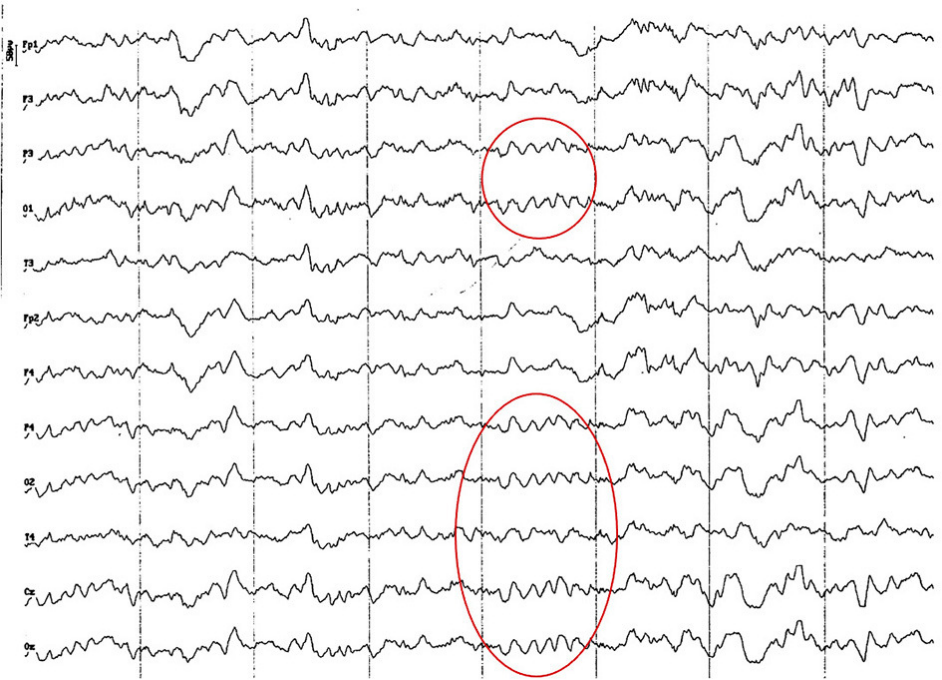
EEG (reference montage) corresponding to a 4-month-old female dog of mix breed and 6.5 Kg of body weight. Division 1 seg/50 μV. It is possible to observe one of the varieties of slow waves that appear paroxysmally at this age: 4 to 6 Hz; in this case, 5 Hz, more noticeable in the occipito-parietal regions (red circle).

**Figure 4:**
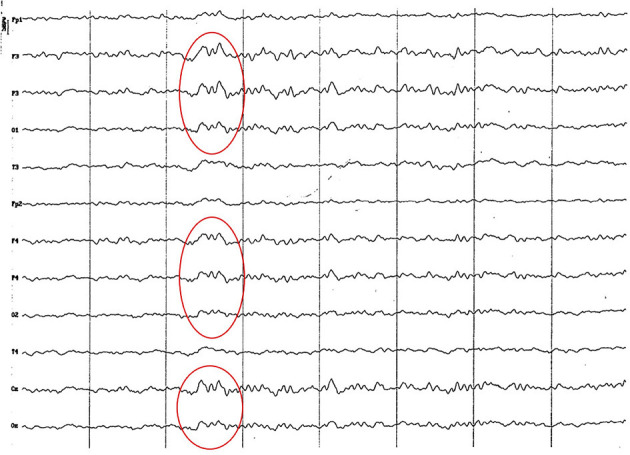
EEG (reference montage) corresponding to a mix breed male German Shepherd, of 5 months of age and 8 kg of body weight. Division 1 seg/50 μV. It is possible to observe another type of slow waves that appear paroxysmally at this age: 3 Hz, more noticeable in the dorsal cortex (red circle), except for the Fp channels. In the same tracing, an outline of alpha activity can be observed, which becomes increasingly visible in the occipito-parietal regions.

Background electrical activity shows a mild diffuse disorganization (frequency mixing), with the theta band as the dominant frequency, with an incipient superimposed alpha activity and paroxysmal slow waves (Figure 2A). Higher amplitude was found in the parieto-occipital region. Temporal (T) and frontopolar (Fp) channels feature a low voltage signal, with predominance of slow waves (3–4 Hz), although with a superimposed fast activity (beta 1). Sharp vertex waves of wide voltage are observed relatively frequently (up to 110 uV) (Figure 2B). Semi-periodic paroxysms of slow waves are also observed, which can be differentiated into 2 different types: waves of 4 to 6 Hz, from 50 to 70 uV (Figure 3); and slow waves of 3 Hz, from 20 to 40 uV (Figure 4). Also, an incipient alpha activity was found (activity from 8 to 9 Hz, in at least 50% of dogs, up to 13 Hz) from 40 to 60 uV, particularly evident in the parieto-occipital region, with an onset frequency of once every 10–15 s (see Figure 4). Intermittent tracing asymmetries were found in half of the animals (higher signal amplitude with a 25–30% of difference between hemispheres), 50% of which corresponded to each cerebral hemisphere. No asynchronies were found.

#### 3.2.2. Group 2 (6 to 11 months of age)

**Figure 5:**
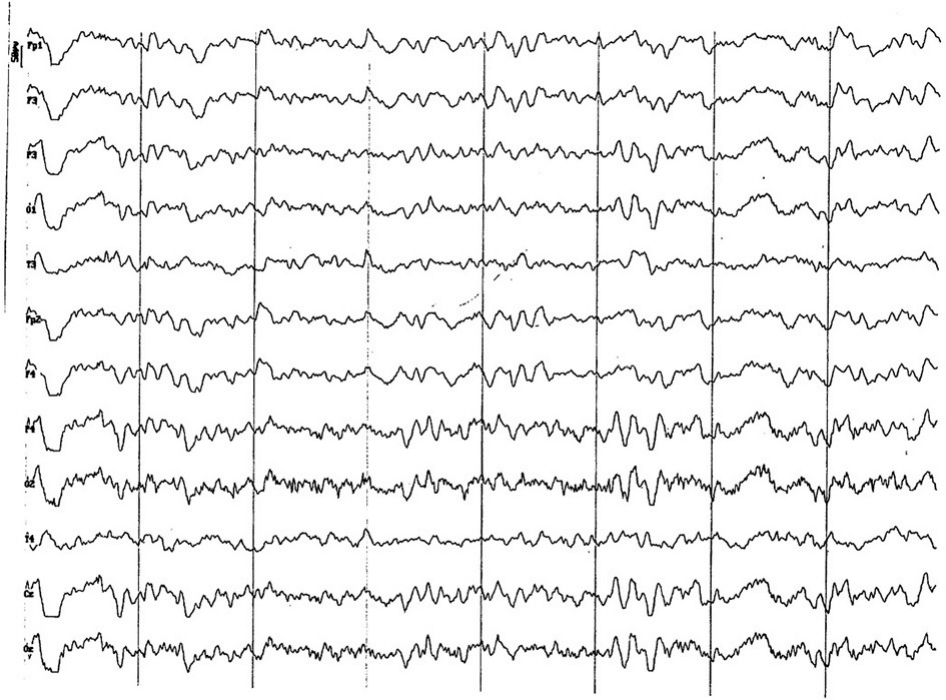
EEG (reference montage) corresponding to a male Miniature Poodle of 10 $\frac{1}{2}$ months of age, body weight 13 kg. Division 1 seg/50 μV. In this period (6 to 11 months of age) the predominance of the theta rhythm can be observed, with outlines of superimposed alpha and slow wave paroxysms. Frontopolar (Fp) and temporal (T) channels have significantly increased the voltage of their electrical signal.

**Figure 6:**
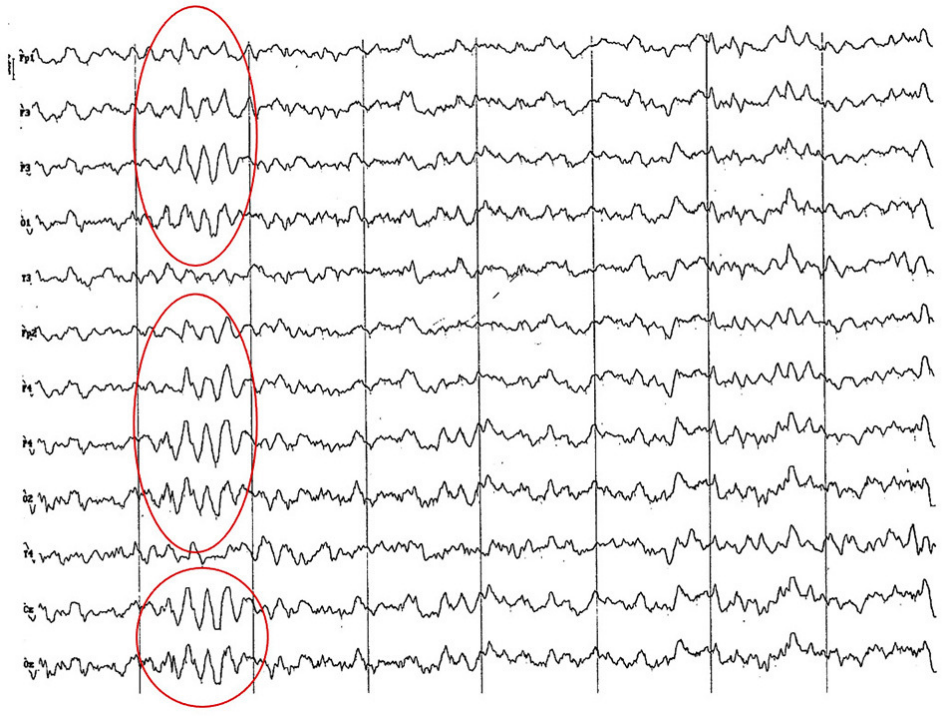
EEG (reference montage) corresponding to a female Cocker Spaniel of 8 months of age. It is possible to observe a paroxysm of slow waves of 5 Hz of high amplitude in the entire dorsal cortex (red circle). Note the dominant slow activity in the temporal channels (T) framed in the theta frequency band. Division 1 seg/50 μV.

**Figure 7:**
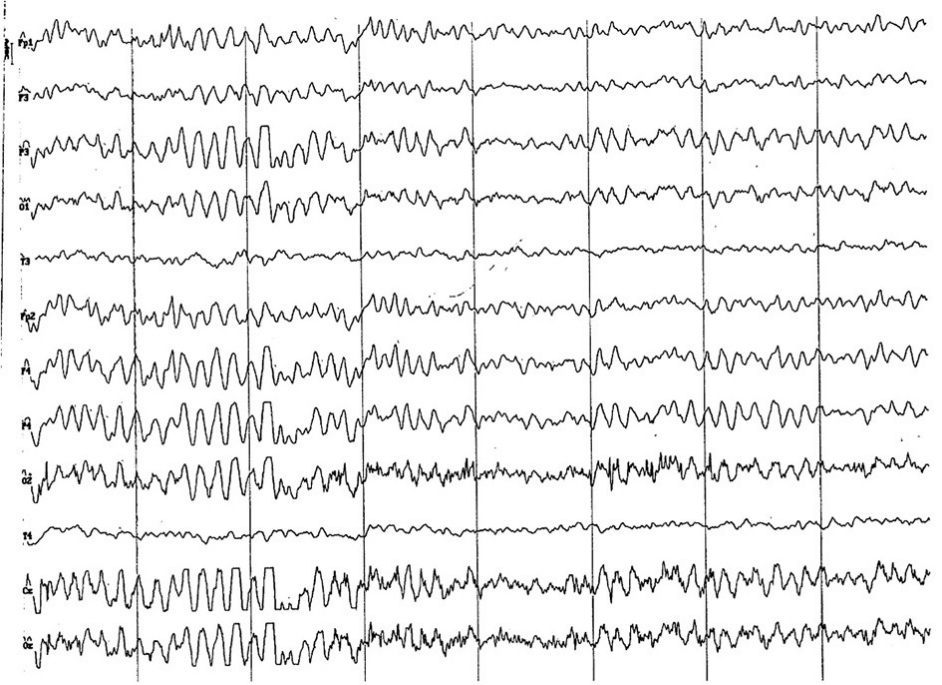
EEG (reference montage) corresponding to a female Miniature Poodle of 9 months of age. Frequent paroxysms of an initial alpha rhythm can be observed, with a frequency, in this case, of 8 Hz. Division 1 seg/50 μV.

Background electrical activity was similar to that of the previous group, albeit the theta rhythm dominance was more evident, always with incipient superimposed alpha activity and paroxysmal slow wave (Figure 5). Higher amplitude was found in the parieto-occipital region. Acute vertex waves continued to manifest. As in the previous group, paroxysmal slow waves were found, but only in the frequency range of 4 to 7 Hz, and about 60 uV (Figure 6). The alpha activity (8 to 13 Hz) continued to appear paroxysmally, always in the parieto-occipital regions, with an amplitude of 30–60 uV (Figure 7). The most noticeable change in this age group was the substantial amplitude increase in frontopolar channels and, in some animals, in the temporal channels. These channels featured slow dominant activity, mainly contained in the theta band (4 to 5 Hz) (see Figure 6).

#### 3.2.3. Group 3 (12 to 17 months)

**Figure 8:**
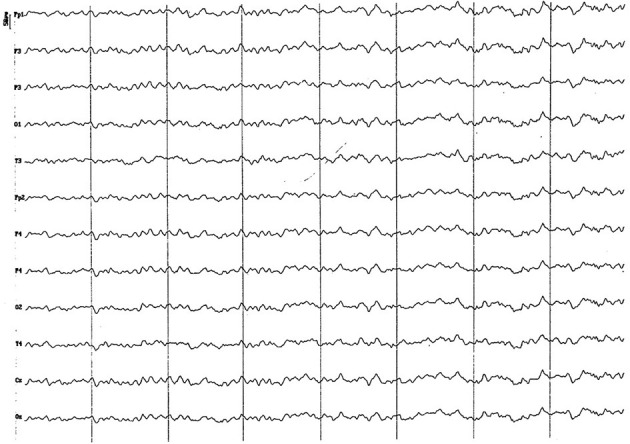
EEG (reference montage) corresponding to a 14-month-old female dog, of mix breed of 13 Kg of Body weight. Division 1 seg/50 μV. In this period (12 to 17 months of age), although the dominant frequency is found in the theta band, the alpha rhythm is almost permanently superimposed on it. Although the temporal channels (T) present an amplitude similar to the rest, they show a clearly lower frequency. Generally, the voltage begins to show a decrease regarding previous periods.

**Figure 9:**
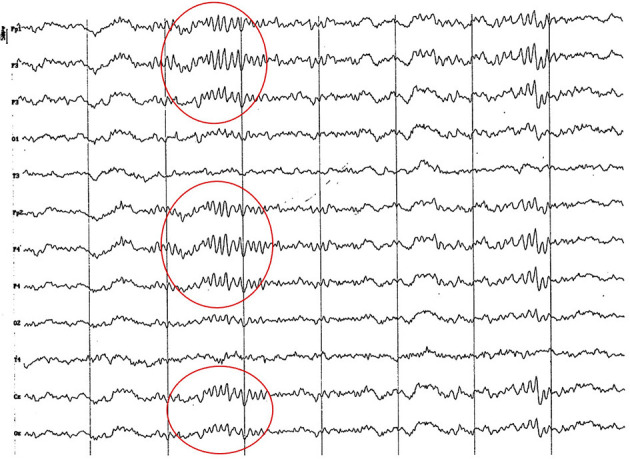
Fragment of EEG (reference montage) corresponding to a Yorkshire Terrier of 15 months of age. Division 1 seg/50 μV. In this brachycephalic animal, higher amplitude alpha activity is observed in the fronto-parietal region (red circle).

**Figure 10:**
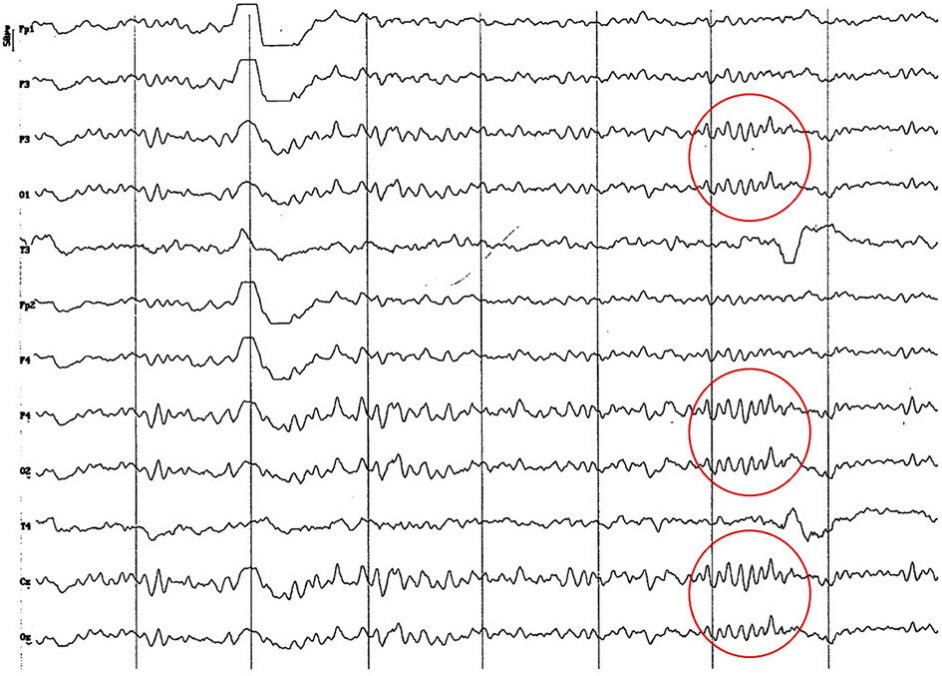
Fragment of EEG (reference montage) corresponding to a female Beagle of 17 months of age. In this mesocephalic animal, higher amplitude alpha activity is observed in the occipito-parietal region (red circle). Division 1 seg/50 μV.

**Figure 11:**
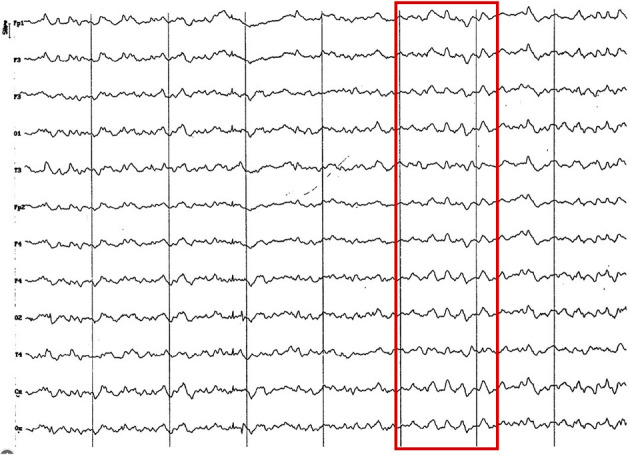
EEG (reference montage) corresponding to a 16-month-old female dog of mix breed, body weight 11.5 kg. It is possible to observe the presence of slow waves, in this case 4 Hz of a lower voltage (red square) to previous age periods. Division 1 seg/50 μV.

The dominant activity in this age group was in the theta frequency band. Nevertheless, an incipient alpha activity started to overlap over this rhythm, particularly in occipital, parietal and frontal regions (Figure 8). At times, this rhythm became the dominant activity. In brachycephalic dogs, the alpha activity was more evident in the fronto-parietal region (Figure 9), while in mesocephalic dogs, in the parieto-occipital region (Figure 10). Paroxysmal slow waves of 4–7 Hz were still observed, but with a smaller amplitude than in the previous groups (25 to 40 uV) and with a less frequent manifestation (Figure 11). The amplitude of the signal recorded in temporal channels and dorsal cortex channels was very similar (see Figure 9), although, temporal leads tracing was significantly slower than previous groups. In this period, a generalized amplitude decrease of the signal recorded begins to be evident.

#### 3.2.4. Group 4 (18 to 23 months of age)

**Figure 12:**
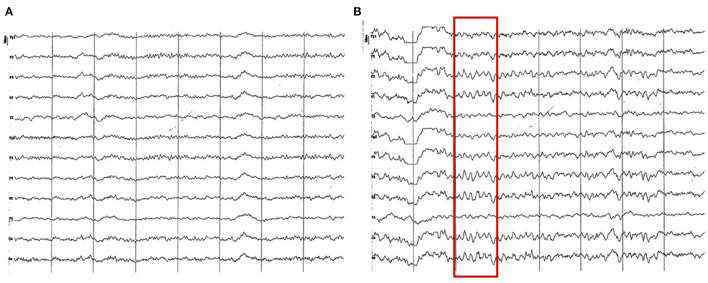
EEG (reference montage) corresponding to a Yorkshire Terrier of 23 months of age **(A)**. Female Miniature Poodle of 19 months of age **(B)**. Division 1 seg/50 μV. **(A)** Note the generalized amplitude decrease, despite being a small breed, which is characteristic of this period (18 to 23 months). Also, observe the intermittent dominance of alpha and theta rhythms. **(B)** Slow wave paroxysms can still be observed, in this case of 6 Hz (red square), although much more sporadically than in previous periods.

The recording was very similar to that of the previous group. Alpha activity became increasingly important, alternating with theta activity (Figure 12A). Paroxysmal slow wave activity of 4 to 7 Hz, 25 to 40 uV, was maintained (Figure 12B), although it became increasingly more sporadic.

One of the most remarkable characteristics in this group was the generalized decrease in the recording amplitude, which was found in all channels, but much more pronounced in temporal and frontopolar channels. In the first cases, the slow dominant activity prevails, which clearly differentiates them from the rest.

#### 3.2.5. Group 5 (24 to 83 months of age)

**Figure 13:**
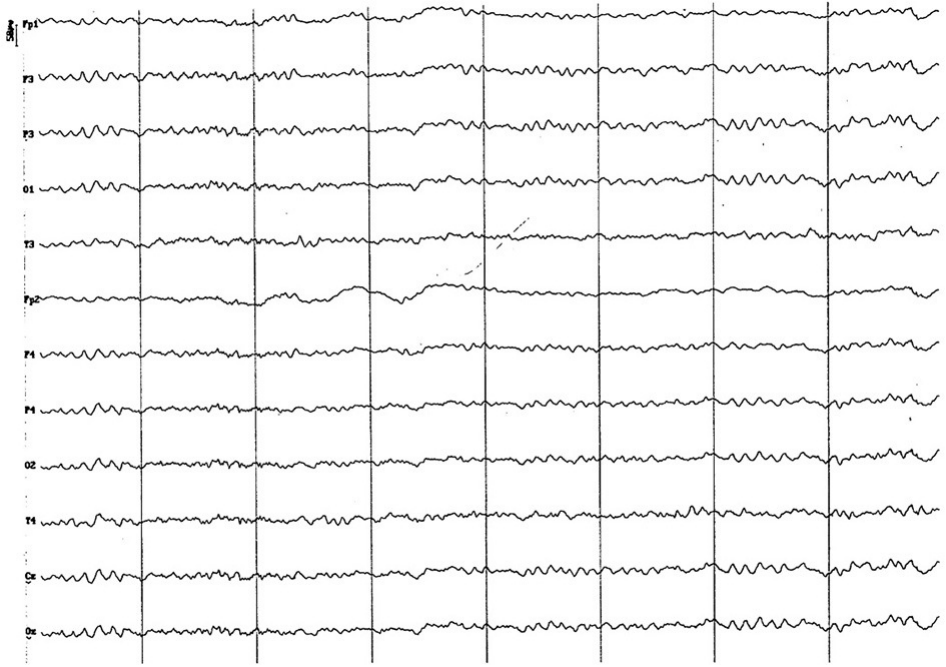
EEG (reference montage) corresponding to a 5-year-old female dog, of mix breed and 16 kg of Body weight. Division 1 seg/50 μV. Features of this period are the low amplitude and permanent presence of the alpha rhythm and the similarity of the electrical signal between the temporal (T) and the other channels.

The electrical activity was very similar to that of the previous group. The only difference consisted in the disappearance of the paroxysmal slow wave activity from 4 to 7 Hz, and a slight attenuation of alpha activity (Figure 13). The recording amplitude in all channels continued to show low values. The difference in frequency of the signal recorded in the temporal channels and the rest was not evident, as in the case of the previous groups.

#### 3.2.6. Group 6 (more than 83 months of age)

**Figure 14:**
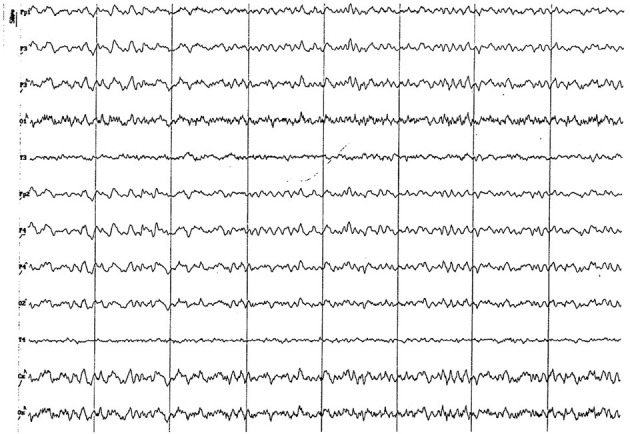
EEG (reference montage) corresponding to a 13-year-old male dog of mix breed and 11 kg of body weight. Division 1 seg/50 μV. An apparent visual increase in the alpha rhythm is observed during this period. Once again, the temporal channels (T) show a lower amplitude than the rest of the channels.

**Figure 15:**
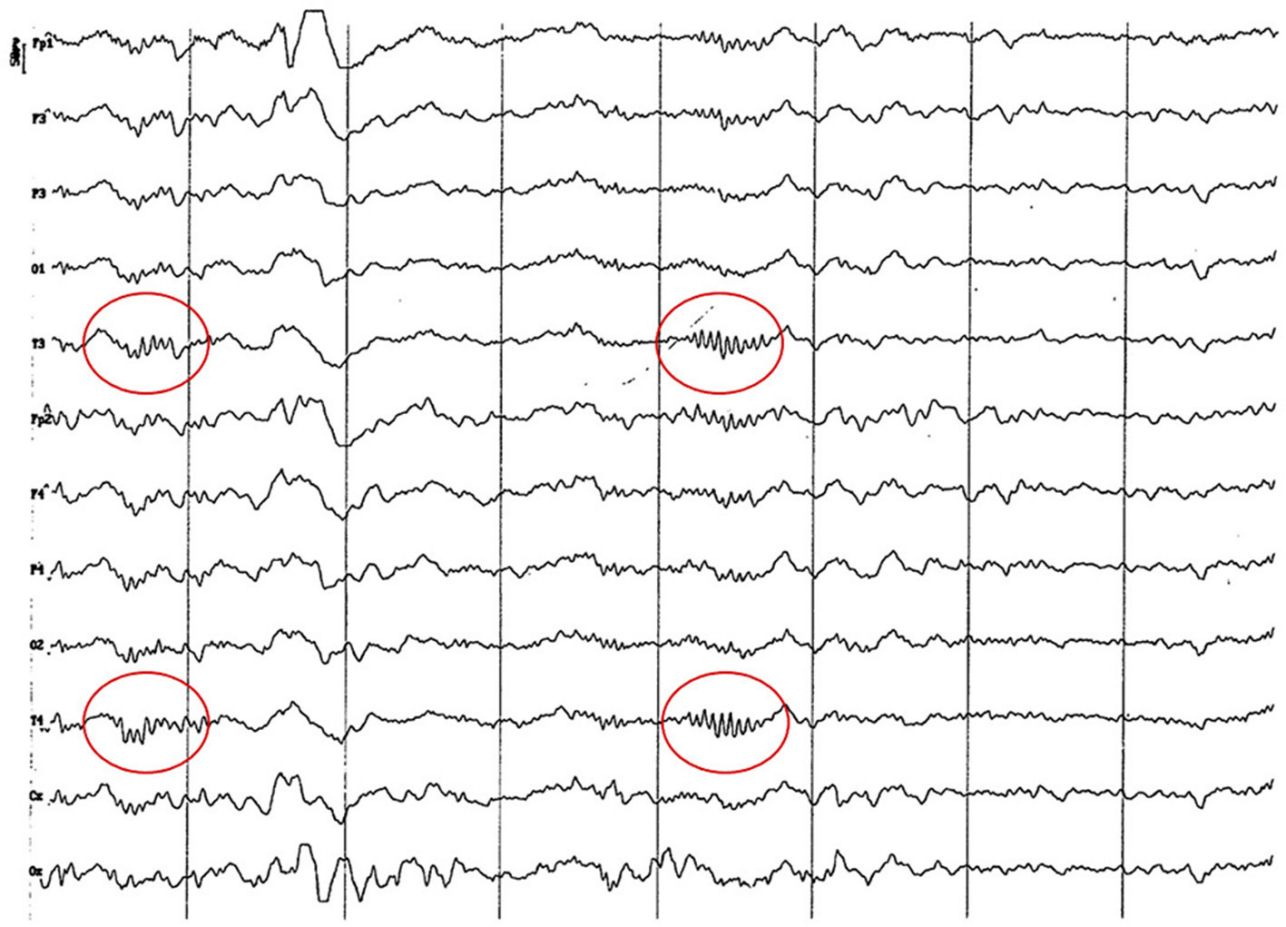
EEG (reference mounting) corresponding to an 8-year-old female dog of mix breed 16 kg of body weight. Division 1 seg/50 μV. probably pseudo-spindle activity (10–12 Hz) can be observed (red circle), particularly noticeable in the temporal channels (T).

No major differences were visible in regard to the previous group, except for an apparent increase in the alpha activity in most of the animals recordings (Figure 14). For the first time possible pseudo-spindles activity were recorded in temporal channels (Figure 15).

### 3.3. Power spectrum analysis

#### 3.3.1. First stage

ANOVA for normalized relative power showed the presence of interactions between age and rhythm factors, and between rhythms and channels. The presence of interactions implies that the individual analysis of each considered factor is not valid without taking into account how the factors influence each other. Since there were no triple interactions, one of the factors may be fixed (at any of its levels) to, then, evaluate how the other two interact freely.

Interaction occurred in temporal channels, where relative power of delta rhythm increased, basically, at the expense of the alpha activity decrease and, to a lesser extent, of theta rhythm. Other important conclusions emerged from the previous analysis: (a) the levels assigned to subdivide the age variable proved to be insufficient, as they could have been concealing certain characteristics by being grouped in intervals of different size; (b) channels pertaining the dorsal portion of cortex (Fp, F, P, O, and central) seemed to have a similar behavior, presenting a strong discrepancy with temporal channels (T); (c) beta rhythm presented an almost constant level throughout all levels of the other factors under study.

#### 3.3.2. Second stage

Beta rhythm was excluded due to its poor participation as a source of variability; age was subdivided into comparable interval groups (0 to 5, 6 to 11, 12 to 17, and 18 to 23 months); channels were regrouped into 2 levels (temporal and dorsal).

ANOVA for normalized relative power for all age groups shows the presence of double interactions. In regard to age/rhythm interaction, comparison of means by general contrast (p < 0.05) makes it possible to determine that values pertaining delta rhythm are different (higher values) from the average of alpha and theta activity for ages 0 to 5 months, 6 to 11 months, and 18 to 23 months, whereas for the 12 to 17 months group (Supplementary Figure 1), differences are not substantial. On the other hand, theta and alpha activity were different (alpha activity higher than theta activity) from one another (Supplementary Figure 1) for 0 to 5, 6 to 11, and 12 to 17 months groups, but no substantial differences were found in the 18 to 23 months group. The general trend for alpha activity values was upward, as for the theta rhythm values. Delta rhythm behavior stood out, with values showing a downward trend until 17 months of age and then showing an inverse trend. This behavior is understood to be the consequence of amplifiers noise. The relative potency of delta and theta frequencies increases as the amplitude of the tracings decreases.

Age/channel interaction exhibited important differences between dorsal and temporal channels in all ages, except for the 0 to 5 months group, where values obtained for both groups of channels were not different from each other. For age of 6–11 months temporal channels values were higher than dorsal channels. For groups 3 and 4 (12–17 and 18–24 months) dorsal channels values were higher than temporal ones (Supplementary Figure 2).

The rhythm/channels interaction was manifested at the level of delta and alpha frequency bands, whose values showed significant differences between dorsal and temporal channels (Delta frequency was higher in temporal channels and alpha activity was higher in dorsal channels). For theta frequency band, the values obtained for both groups of channels were not different from one another (Supplementary Figure 3).

ANOVA for normalized relative power for age groups 24 to 83 months (adults) and more than 84 months (older dogs) which were analyzed separately, only showed a rhythm effect. There were no double or triple interactions. In other words, in dogs older than 2 years of age, only a variation on the power spectrum composition was found in terms of percentage distribution of electroencephalographic rhythms. The analysis of its behavior by comparison of means (Tukey, *p* < 0.05) indicated that, after 2 years of age, delta rhythm is significantly higher from alpha and theta activity, while the two latter do not differ much between each other (Supplementary Figure 4). We consider this delta behavior as a consequence of an amplifier noise.

Results obtained through visual analysis were consistent with those of the statistical impact analysis that age, channels, and electroencephalographic rhythms have on relative power.

## 4. Discussion

In order to establish the EEG maturation process through visual analysis, we have selected 3 paramount characteristics: (a) the presence or absence of slow waves from 4 to 6–7 Hz; (b) comparisons of electrical activity recorded in temporal and dorsal cortex channels; and c) visual increase of alpha activity.

In the maturation process of the EEG in dogs, the presence of slow waves of 4 to 6–7 Hz is a key element. The onset of such waves has been described between 5 and 7 weeks of age and their disappearance between 5 and 6 months of age (7, 8, 10, 19, 20). It is likely that the development of cortical neurons and the thalamus-cortical influence produced up to the first month of age (3, 6, 8, 25), but which last longer, up to 10 weeks of age (6–8, 26), is somehow connected to their onset. On the other hand, the strengthening process of ideal synaptic connections and the elimination of unnecessary ones, which occurs approximately up to 4 months of age in dogs (27–29), influences the disappearance of slow waves. Nevertheless, we have found them in all age groups up to 23 months of age. We consider this to the fact that the final postnatal development phase continues for life, and it is characterized by the stability and strengthening of established synapses (27–29).

Our findings suggest that EEGs rhythmic patterns in dogs remain constant and do not present crucial variations from the age of 2 years. From that age onwards, we consider that the brain electrical activity is mature. This includes adult, mature, senior, and geriatric dogs (30). A significant contribution to this fact is that temporal electrodes show a very similar activity to other electrodes. Alpha activity is consolidated as the dominant frequency, while slow waves and differences between the dorsal and temporal channels disappear.

In humans, when maturing, slower frequencies decrease and higher frequencies increase (31). This change from low to high EEG frequencies is a characteristic hallmark of brain maturation (32). We found a two-way interaction during the period between birth and 23 months of age between electroencephalographic rhythms, recording channels and age when analysis of relative power behavior was done. Values of alpha and theta rhythms showed a significant increase from 6 months of age. Alpha activity, although increasing its relative values, was still substantially lower than theta rhythm up to 17 months of age; from this age on, values in both were similar. Delta rhythm values exhibited a progressive decrease until 17 months of age; however, from 18 month of age, they showed a progressive increase. To interpret this phenomenon and correlate it with the visual analysis of the EEG, it is necessary to consider that power spectrum shows all the frequency components of the complex analyzed signal. Therefore, all extracerebral signals affecting the lower end of the relative the power spectrum (e.g., body movements) will be represented in the delta band. Thus, the increased statistical value of delta rhythm had no correlation with visual analysis.

Our assessment about EEGs maturation age differs markedly from those made by other authors, who have established it between 6 and 7.5 months of age (9, 10). This discrepancy is attributable to the use of temporal electrodes, which record the electrical activity of the rhinencephalic cortex. Maturation periods of neopallium and rhinencephalon and their electrical rhythms differences were manifested in the features of electrical activity recorded in temporal and dorsal cortex channels.

In dogs, the development and maturation of rhinencephalic structures have not been cleared yet. However, our findings suggest that it may be established between 6 and 11 months of age when a substantial increase of amplitude in the temporal channels. Statistical analysis shows at this age range significant amplitude differences between the temporal and dorsal channels, with the former ones reaching the highest values.

Through visual analysis of EEG recordings, we identified five development stages in dogs, which coincided with the preselected age groups. Statistical analysis of relative power behavior, particularly with regards to the age/rhythm interaction, support this division.

The period prior to that signaling EEG maturation spans from birth to 23 months of age and can be subdivided into 4 stages, with different features. First stage (0 to 5 months of age) visual analysis is characterized by the mixture of frequencies (predominantly slow ones) and by the low amplitude recorded in frontopolar and temporal channels. However, statistical analysis did not find differences between temporal and dorsal channels. Probably low voltage of Fp channels reduces the average value of dorsal channels, thus matching the average of temporal channels. ANOVA did not find age to be significant factor among the dogs of this group (*p* < 0.05) although some authors have suggested important differences at this stage (2, 7–10, 19, 26). Therefore, there are not differences which validate its division into smaller subgroups. We believe that, for the purpose of clinical application, it is not necessary to subdivide this group, at least in animals older than 45 days, considering that our study did not include any individual under this age.

For second stage (6 to 11 months of age) slow frequencies were still dominant, but theta rhythm is predominant in the visual analysis. The alpha activity began to appear incipiently, particularly in parieto-occipital regions. But the foremost event was the amplitude increase in frontopolar and temporal channels. Statistical analysis supports these observations. We interpret this fact as a maturational process of limbic cortical domains, which is probably closely related to behavioral modifications manifested in this period. This age corresponds to the first stage of behavioral maturation in dogs (33, 34), in which the characteristics concerning the personality and character of the individual begin to be defined.

On visual analysis, the third stage (12 to 17 months of age) showed a very similar pattern to that of the mature adult, yet with differentiating aspects. Theta rhythm was still the dominant frequency, but alpha activity remained frequently superimposed. It was not visually possible to identify the delta rhythm when the animal was arising from sedation and the slow waves of 4 to 6 Hz, although still significantly present, decreased their amplitude considerably. The statistical analysis support these observations since it showed an important decrease of delta rhythm values, matching the theta rhythm values. Alpha activity increased its relative values, but was still notably lower than delta and theta rhythm.

The fourth stage (18 to 23 months of age) was characterized by voltage decrease of the signal recorded and an increase of the alpha activity on visual analysis. Nevertheless, slow waves were still found intermittently, from 4 to 6–7 Hz with low amplitude. Statistical analysis showed the definite impact of age/rhythm interaction, which remain in the rest of the analyzed ages, becoming one of the hallmarks of the adult animal: alpha activity values match those of theta. In humans, theta rhythm is mainly found in infants, and it continually decreases with age (35). Theta rhythm areas are frequent on posterior regions in children between 7 and 10 years of age. This phenomenon may be a precursor of alpha rhythm in adults, and may be connected to maturation, overlapping areas implied in the generation of a lower frequency alpha rhythm (36).

The impact of the age/channel and rhythm/channel interaction on relative power behavior does not strictly adjust to the 5 age groups described and will be considered separately.

The impact of the age/channel interaction between 12 and 23 months of age showed a substantial change in relative power behavior of temporal and dorsal channels, with this latter reaching the highest values. This fact is due to the generalized amplitude decrease and appears in this group and consolidates from the 24 months of age. This voltage reduction differentially affects temporal channels, probably due to the anatomy of the region where they are placed.

Although the third and fourth stages were very similar to mature dog EEG recording, we considered them different based on the discrepancy between temporal and dorsal cortex channels on the visual analysis. The period between 12 and 23 months of age included the second stage of the behavioral maturation in dogs, in which the consolidation of the features concerning personality and character of the individual is observed (33, 34). It is likely that the synapse neoformation processes (synaptogenesis) and persistent changes in functional properties of neuronal groups (synaptic plasticity) (37), are involved in behavioral changes found between 6 and 23 months of age, and somehow linked to electrical features of the limbic region.

The rhythm/channel interaction analysis indicated that, for any of the studied ages, the composition of the frequency spectrum is different for temporal and dorsal channels. Alpha activity was higher for dorsal channels and delta rhythm was higher for temporal channels. Amplitude average of temporal channels is considerably low for four ages groups. Therefore, delta activity shown by the statistical analysis is artificially high, being theta the dominant frequency band on visual analysis. This finding is coherent with bibliographic information about rhythmic slow electrical activity (RSA), typical of limbic cortical areas. This activity appears modulating not only normal synaptic transfer, but also long-term changes in the synapse strength (7). In addition, this fact matched visual analysis of the signal obtained in temporal channels.

No differences have been found among the EEG of studied animals older than 2 years of age. The oldest group (more than 83 months), with an average of 8.5 and peaks of 11 and 13 years of age, showed no differences with animals of 24 to 83 months of age. Decrease in the recording amplitude in “old” animals stated in the cited bibliography (9) did not represent a specific feature of this group. Voltage decrease occurs substantially from 18 months of age and is probably due to the increase of skull thickness and surrounding muscles. These structural changes subdue the brain's electrical signal. These findings agree with statistical analysis, since from 2 years of age the interaction among analyzed factors disappears, showing the individual effect of the EEG rhythm factor. Thus, for any age older than 2 years, and any channel, alpha and theta activity will have similar contribution to power spectrum. This statistical data was consistent with the visual analysis, in which a similar electrical activity was observed on temporal and dorsal channels from 24 months of age. By the first time we had seen some graphoelements compatible with a possible pseudo-spindle which origin might be the hippocampus and the mechanism underlying this feature still unknown (38, 39). It differentiates from alpha rhythm (Rhythm at 8–13 Hz inclusive during wakefulness over the posterior regions of the head, generally with the higher amplitudes over the occipital areas) and sleep spindles (Train of distinct waves with a frequency at 11–16 Hz with a duration of >0.5 seg and generally with higher amplitude over the central areas) as pseudo-spindles seems to be recorded with higher amplitude at the temporal leads arising from hippocampus and having a slow rhythm (38–40).

All information above refers to the visual analysis of the EEG recording and the statistical analysis of the relative power behavior. However, interhemispheric coherence analysis studies have reported that, even in the period of electrical maturity (2 years of age), differences persist between the signal recorded in temporal and dorsal cortex channels. These differences result from their origin, modulation and transfer over distance (18). Such functional differences are simply the reflection of large structural and morphological differences among isocortex and allocortex. Local nature factors acquire great relevance in the electrical activity of the limbic cortex. This is supported by evidence of the theta hippocampal activity origin, especially in terms of its cellular bases within pyramid neurons from CA1 and CA3, as well as its relation to a septum-induced disinhibitory process (41–43). These features might increase susceptibility of the limbic cortex to some epileptic disorders (44, 45).

## 5. Conclusions

According to the findings in the present study, maturation of brain bioelectrical activity in dogs is a gradual process and the EEG features of adult animals start showing at 12 months of age, but stabilize after 24 months of age based on the EEG visual analysis and the statistical analysis of spectral power behavior. In this process, the evident differences in the development and maturation of the neopallium and rhinencephalon play a crucial role. The elements to be considered in order to visually identify the EEG maturational periods in dogs are: (a) the presence or absence of slow waves of 4 to 6–7 Hz, (b) homogeneity of signal recorded in the totality of EEG channels and (c) the dominance of alpha rhythm. These features are easily recognizable in the visual analysis of the EEG recording.

It is relevant to know background and normal transient activities of each developmental period in order not to misinterpreted certain graphoelements as abnormal (e.g., slow waves that feature the immature EEG), which would result in wrong decisions regarding diagnosis, prognosis and/or treatment of patients under a brain maturation process with intracranial pathologies.

## Data availability statement

The raw data supporting the conclusions of this article will be made available by the authors, without undue reservation.

## Ethics statement

The animal study was reviewed and approved by Comisión Institucional para el Cuidado y Uso de Animales de Laboratorio (CICUAL) Facultad de Ciencias Veterinarias-Universidad de Buenos Aires. Written informed consent was obtained from the owners for the participation of their animals in this study.

## Author contributions

All authors listed have made a substantial, direct and intellectual contribution to the work and approved it for publication.
