# Supplementary material for: Electroencephalographic features of the developing brain in 72 dogs under xylazine sedation: a visual and statistical analysis
Source: Front Vet Sci. 2023 Jun 29;10:1150617. doi: 10.3389/fvets.2023.1150617 (PMC10339384; doi:10.3389/fvets.2023.1150617)
Supplement: Supplementary file 1 [file Data_Sheet_1.PDF]

Supplementary Graphics and Figures

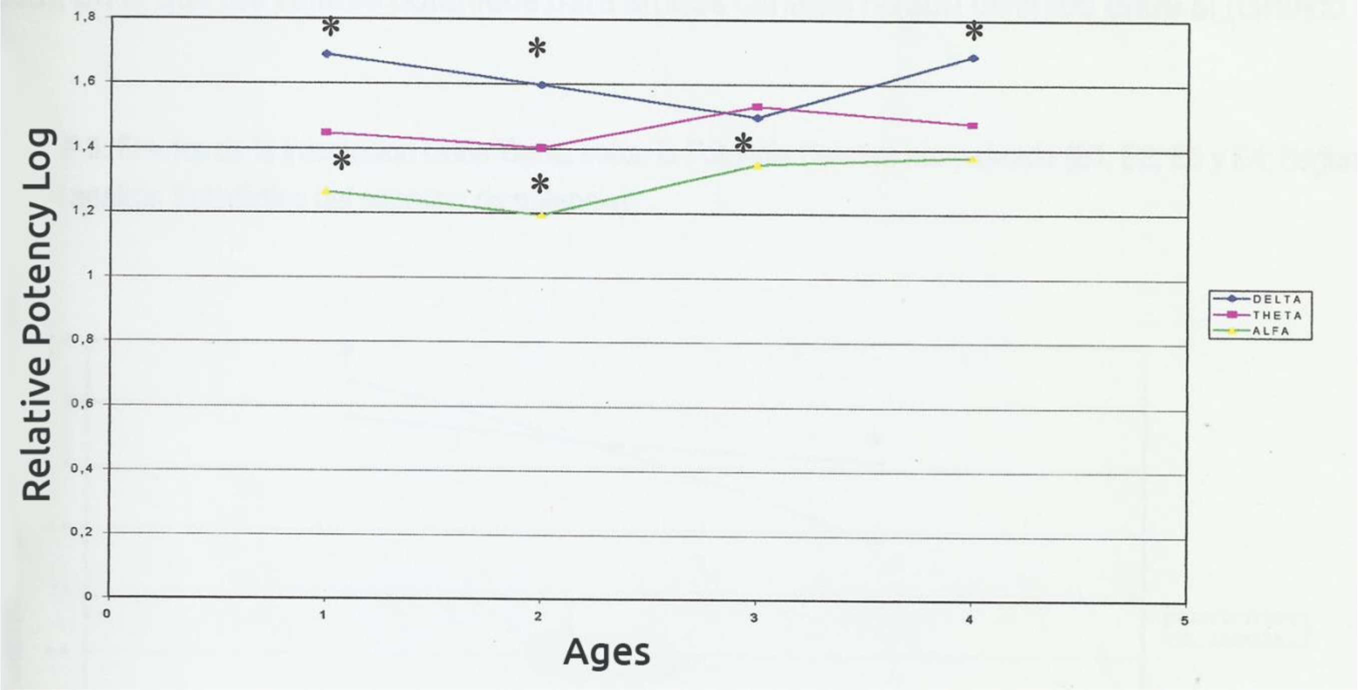

Figure 1. Effects of interaction Age/Channel over the normalize relative potency in groups 1 (0-5 months), group 2 (6-11 months), group 3 (12-17 months) and group 4 (18-23 months). Blue line delta activity, pink line theta activity and green line alfa activity.

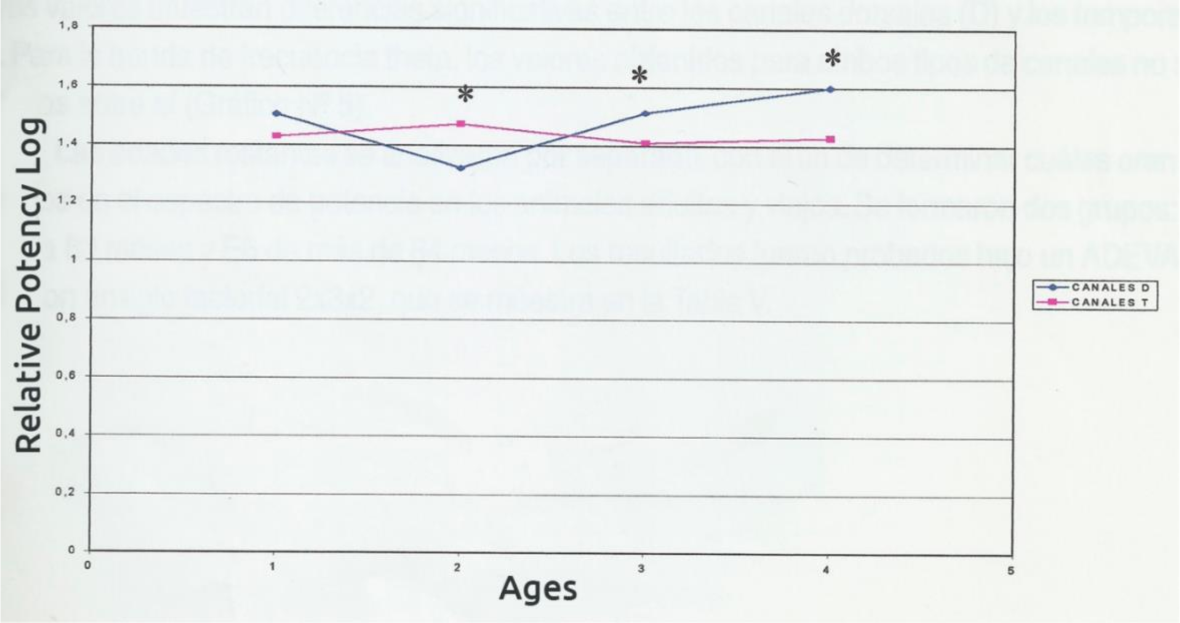

Figure 2. Effects of interaction Age/Channel over the normalize relative potency in groups 1 (0-5 months), group 2 (6-11 months), group 3 (12-17 months) and group 4 (18-23 months). Second stage of power spectrum statistical analysis. Blue line dorsal Channels, pink line temporal channels.

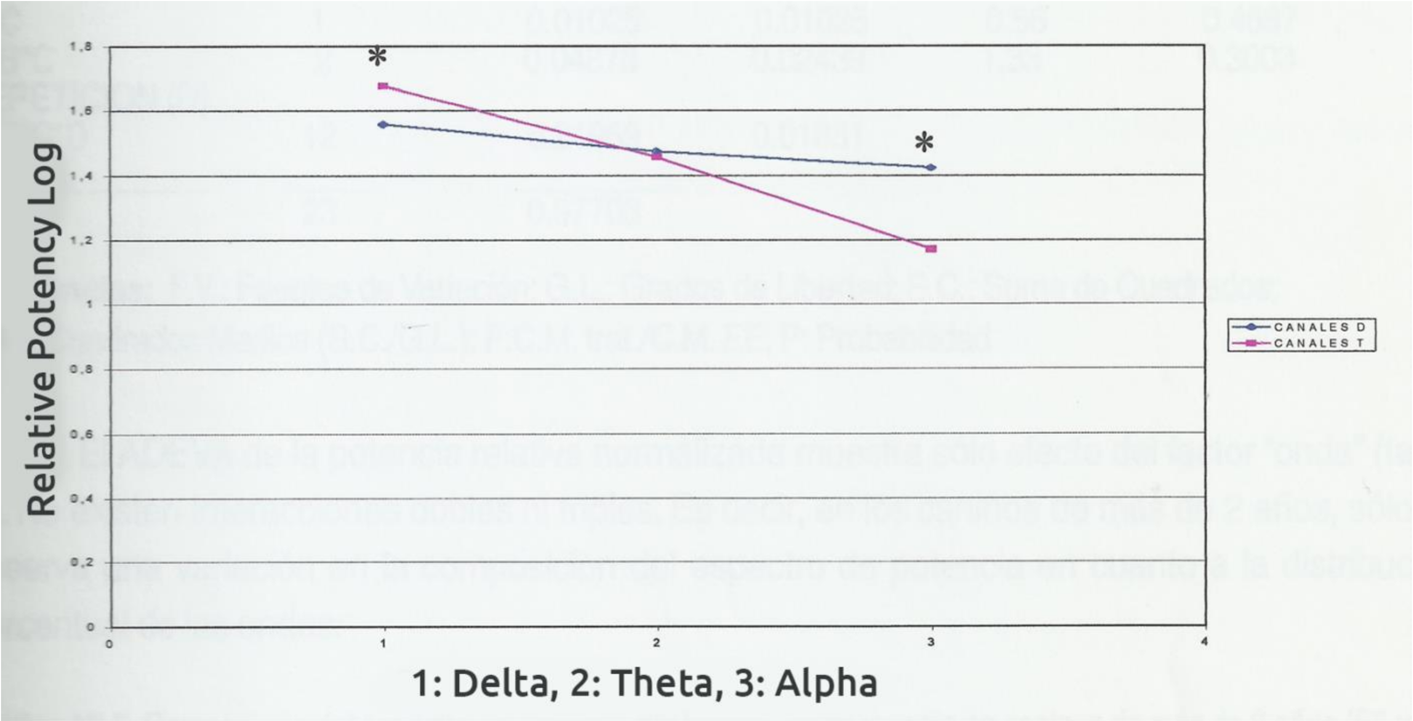

Figure 3. Effects of interaction rhythm/channels over the normalize relative potency in groups 1 (0-5 months), group 2 (6-11 months), group 3 (12-17 months) and group 4 (18-23 months). Second stage of power spectrum statistical analysis. Blue line dorsal Channels, pink line temporal channels.

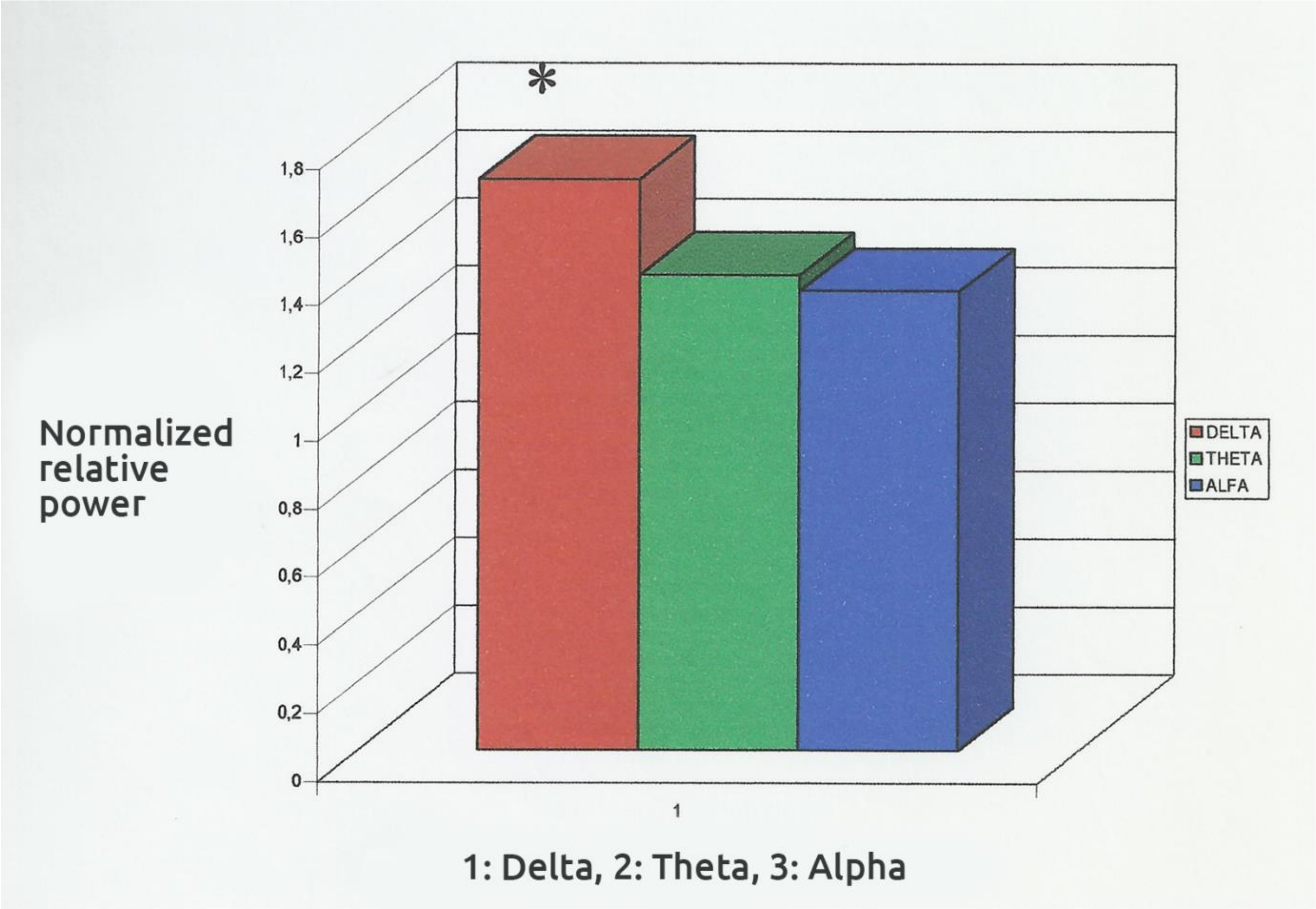

Figure 4. Composition of power spectrum frequency bands in dogs older than2 years. Groups 5 (23-83 months) and group 6 (> 83 months). Second stage of power spectrum statistical analysis. Red Column delta rhythm, green column theta rhythm and blue column alpha rhythm.
